# Integrating geological archives and climate models for the mid-Pliocene warm period

**DOI:** 10.1038/ncomms10646

**Published:** 2016-02-16

**Authors:** Alan M. Haywood, Harry J. Dowsett, Aisling M. Dolan

**Affiliations:** 1 School of Earth and Environment, University of Leeds, Woodhouse Lane, Leeds, West Yorkshire LS2 9JT, UK; 2 Eastern Geology and Paleoclimate Science Center, US Geological Survey, 12201 Sunrise Valley Drive, Reston, Virginia 20192, USA

## Abstract

The mid-Pliocene Warm Period (mPWP) offers an opportunity to understand a warmer-than-present world and assess the predictive ability of numerical climate models. Environmental reconstruction and climate modelling are crucial for understanding the mPWP, and the synergy of these two, often disparate, fields has proven essential in confirming features of the past and in turn building confidence in projections of the future. The continual development of methodologies to better facilitate environmental synthesis and data/model comparison is essential, with recent work demonstrating that time-specific (time-slice) syntheses represent the next logical step in exploring climate change during the mPWP and realizing its potential as a test bed for understanding future climate change.

## Abstract (web summary)

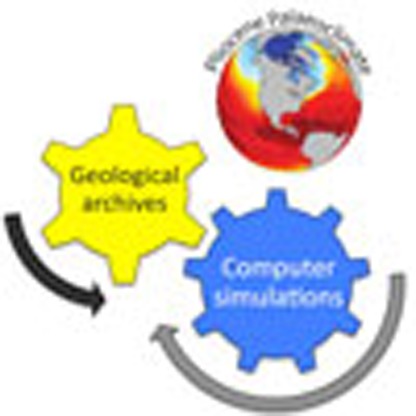
The mid-Pliocene Warm Period (mPWP), analogous to future climate conditions, is considered a test-bed for the predictive capability of climate models. Here, Dowsett *et al*. review our understanding of the mPWP and discuss recent and future advances in the context of proxy data/model integration.

The Earth is experiencing an unequivocal warming unprecedented over decades to millennia^1^. The geological record and simulations of past climate provide scientists with the opportunity to understand how Earth system processes operate outside the observational or instrumental record, and are fundamental to our understanding and ability to accurately project future climate and environmental change^2^. The Pliocene Epoch (5.33–2.58 Ma) is particularly attractive with respect to understanding future climate due to many shared similarities with regards to Earth's physical characteristics.

Both palaeoenvironmental reconstructions and climate modelling have been pivotal in increasing our understanding of climate and its importance during the mid-Pliocene Warm Period (mPWP: 3.264–3.025 Ma; also referred to as the ‘Pliocene Research, Interpretation and Synoptic Mapping (PRISM) interval'). During warm intervals of the Pliocene, atmospheric CO_2_ concentration is estimated to have ranged between 350 and 450 p.p.m.v. (refs 3, 4, 5, 6, 7, 8). This is in contrast to the known pre-industrial concentration of 280 p.p.m.v. On the basis of palaeoenvironmental reconstruction, Pliocene surface temperatures over land and oceans were elevated with respect to the pre-industrial^9^,^10^. Climate model estimates indicate that the global annual mean surface temperature was 2.7–4.0 °C higher^11^. A combination of modelling studies and geological data has shown, for example, that during the mPWP the hydrological cycle was enhanced^11^, ice sheets were smaller^12^,^13^, sea level was higher^14^,^15^, forest cover was expanded and arid deserts contracted^16^.

Many parallels can be drawn between the mPWP and observed trends in modern climate, as well as in future climate projections, due to similar continental configurations, land elevations and ocean bathymetries^17^. Many terrestrial and marine flora and fauna are extant, increasing the validity of approaches that attempt to reconstruct the environment using modern relationships between climate and biogeography^18^,^19^. The mPWP^20^,^21^ (Fig. 1) provides arguably the best pre-Quaternary example of the synergy between numerical models and proxy data, which has ultimately led to an enhanced understanding of this time period.

Since the late 1980s, scientists have used palaeoenvironmental data to systematically reconstruct ocean temperatures, vegetation cover and ice extent, providing a fundamental understanding of the warm nature of the mPWP. Proxy-based reconstructions alone are unable to tell us about mechanisms for warmth and what might be the main climate drivers of sustained globally higher temperatures during the mPWP. The realization of the potential benefit of including climate modelling led to the design of digital data sets specifically for climate models^21^.

Over the last 25 years, the relationship between models and data has evolved and become progressively intertwined and complex. The increasingly sophisticated nature of geological reconstruction and climate modelling techniques has driven a continuous process of innovation in combining data and modelling approaches for understanding the mPWP and what it may be able to tell us about future global change^11^,^22^,^23^,^24^.

Our review begins with an overview of mPWP palaeoenvironmental reconstruction and then discusses the main palaeoclimate modelling efforts focused on this interval. We show how data have progressed from simply providing boundary conditions for numerical models to a means of assessing the efficacy of model simulations of the mPWP climate (for example, data/model comparison). The use of data as a benchmark highlights the complexity of the relationship between data and models, as science demands more statistical rigour and a greater understanding of the potential errors/uncertainty associated with both modelling and data generation. We also use the case study of temperature stability in the tropics to highlight the complexities of this relationship. Finally, we examine recent developments in understanding the mPWP.

## Historical perspective of palaeoenvironmental reconstruction

Zubakov and Borzenkova^25^ were the first to propose that the climate of their Pliocene Optimum (4.3–3.3 Ma) could be considered a past analogue for the mid-Twenty first century when atmospheric CO_2_ concentrations would reach double their pre-industrial values. They pioneered the earliest efforts towards construction of regional Pliocene palaeoclimate conditions based on the palaeontology of more than 20 continental sections and available marine core sequences. From these data they reconstructed 30 Miocene and Pliocene ‘superclimathems' defined as cycles of 100,000 to 300,000 years with amplitudes of at least 4 to 5 °C.

With the *then* generally accepted knowledge that the Pliocene was a time of warm, equable climate, and with growing concern over potential impacts of future global warming as a backdrop, the need for a more precise and less anecdotal assessment of Pliocene climatic conditions became clear. In 1988, the US Geological Survey endeavoured to reconstruct the palaeoenvironment during the mPWP via PRISM. Using lessons learned from earlier efforts to reconstruct the surface of the Ice-Age Earth^26^, PRISM and its collaborators developed a large-scale data collection project that has grown in size and scope over the past 25 years (Box 1). PRISM remains the only global-scale *synoptic* reconstruction of the Pliocene. Data are produced from a global distribution of localities; however, work is concentrated on a focused stratigraphic interval (currently 3.264–3.025 Ma)^17^ (Box 1).

Such an endeavour to understand one stratigraphic interval (time slab) is fundamentally different from efforts that have gone into developing long time series at single locations. When time series intersect the mPWP, we improve our ability to understand the dynamic development and evolution of environment and climate (see also next section). These approaches (time slab and time series) are not mutually exclusive. Rather, each benefits from the perspective only available through the other.

**Figure 2:**
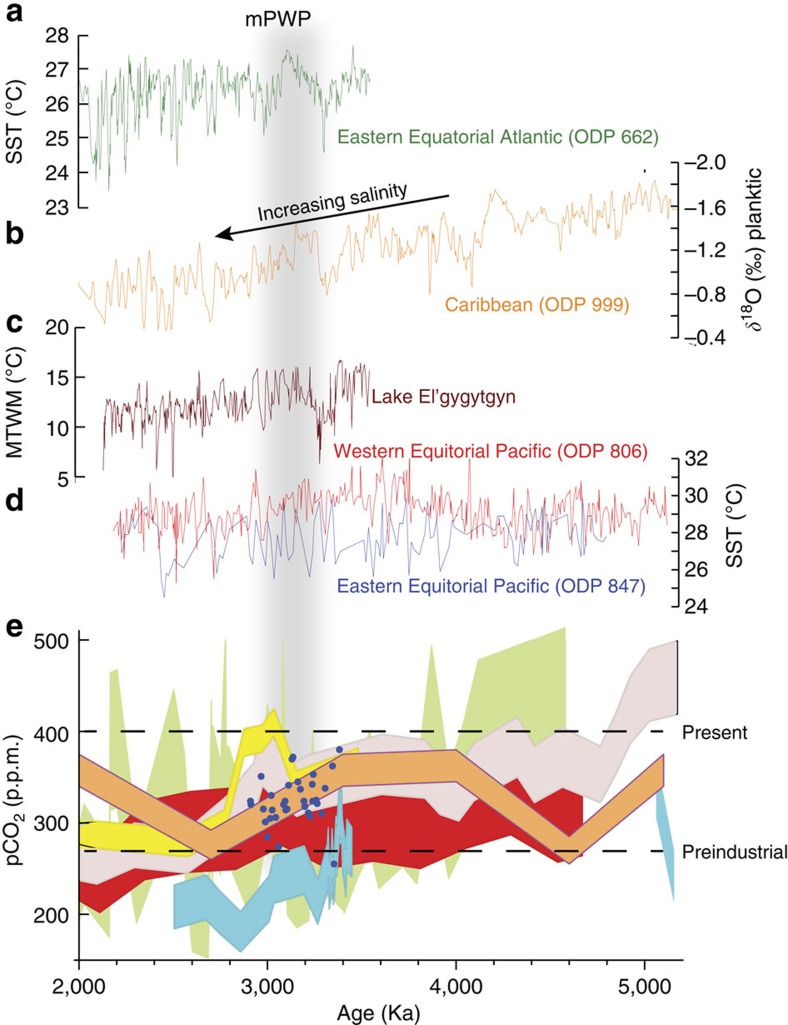
Sample time series analyses. Time series illustrating commonly used palaeoenvironmental proxies. Vertical grey band represents position of mPWP. (**a**) Equatorial Atlantic SST based on alkenone unsaturation index^44^. (**b**) Caribbean Sea oxygen isotope record^111^ showing increasing salinity due to shoaling of the Central American Seaway. (**c**) Terrestrial record of the mean warmest month temperature from Lake El'gygytgyn^56^. (**d**) Equatorial Pacific SST records based on Mg/Ca palaeothermometry^51^. (**e**) Estimates of Pliocene atmospheric CO_2_ with pre-industrial and present day levels (horizontal dashed lines) for comparison; dark blue dots, *δ*^13^C (ref. 3); green band, *δ*^11^B (ref. 7); pink band, alkenone^5^; red band, alkenone^6^; orange band, stomata^4^; yellow band, *δ*^11^B (ref. 5); blue band, Ba/Ca^59^; Modified from ref. 50.

The tools used by the palaeoclimate community have changed over time, and researchers continue to use the most sophisticated techniques available for reconstruction (Fig. 2). Proxies have been developed to reconstruct different aspects of the environment; however, surface ocean and land temperatures are by far the primary variables reconstructed. Techniques include quantitative analysis of faunal and floral assemblages and stable isotopic composition of carbonates and biomarkers. All have strengths and limitations, but each plays an important role in our conceptual understanding of the mPWP.

Stable isotopes of oxygen have been used to estimate palaeotemperatures, and indirectly ice volume, since the pioneering work of Emiliani^27^. Over time they have become the multitool of palaeoceanographic inquest. Shackleton *et al*.^28^ generated the first long benthic *δ*^18^O time series spanning the Pliocene, placing limits on potential changes in the cryosphere, the stability of Antarctic ice and sea level. The development of an isotopic composite standard reference section, LR04, is possibly the most unifying development to date in pre-Pleistocene palaeoceanography^29^. With the LR04 composite, researchers are able to correlate remote sequences to a standardized section tied to orbital chronology. This provides the community with a high-resolution stratigraphic framework within which the temporal and spatial aspects of climate change during the mPWP can be analysed.

Primary temperature proxies commonly utilized for mPWP reconstruction include the following: Mg/Ca palaeothermometry, the alkenone unsaturation temperature proxy 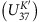, the TetraEther index (TEX_86_) and quantitative analysis of faunal and floral assemblages. These palaeotemperature proxies measure different aspects of temperature by sampling the marine environment at different times of the year as well as at different water depths. While this can provide much insight into the thermal structure of the water column, variations in the timing of production in the past can cause seasonal biases in our interpretations. Likewise, changes in preferred depth habitats would skew palaeoenvironmental reconstruction. Estimates are complicated by effects unique to different signal carriers and methods. For example, the Mg/Ca method depends on assumptions about the composition of seawater at the time calcite was precipitated and can be complicated by post-depositional processes such as dissolution. Faunal assemblage techniques require the assumption of stationarity of ecological preferences and have upper and lower limits based on calibration to present day conditions. 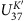, based on extraction of organic molecules (ketones synthesized by haptophyte algae) from sediment, is not affected by seawater chemistry but has an upper limit of ∼28 °C. TEX_86_ (based on the relative distribution of membrane lipids produced by Crenarchaeota), such as 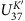, is not directly affected by seawater composition, has an upper limit close to 38 °C, but can sometimes record subsurface conditions and be complicated by terrestrial input^30^,^31^,^32^,^33^,^34^,^35^,^36^,^37^,^38^,^39^,^40^. The use of multiple proxies is of great benefit for gaining more robust and detailed estimates of the palaeoenvironment as long as the relative limitations of various techniques are recognized.

On the basis of these proxy techniques, past studies^41^ have summarized mPWP oceans as being characterized by a reduced meridional SST gradient, potentially driven by enhanced ocean heat transport^42^. High-latitude regions were warmer than those of today, and this warming was at least in part related to changes in sea-ice cover. SST increases in the North Atlantic appear to be particularly pronounced (Fig. 5; see also refs 43, 44, 45, 46). In the circum-Antarctic region, the Polar Front Zone was expanded but displaced towards the continent, and sea ice was greatly reduced^18^,^47^. Subtropical gyres in all oceans were displaced towards the poles.

To highlight the benefit of using multiple proxies, we focus on efforts to understand the tropics, both in terms of data and climate modelling studies (see sections below for further discussion with regard to data/model comparison).

A clear pattern of tropical SST warming has never been evident in the PRISM reconstructions outside of upwelling regions^17^,^18^,^20^,^35^,^42^,^48^. Tropical upwelling regions are fed by intermediate depth waters that originally form and sink in higher latitudes. Since high-latitude SST reconstructions indicate substantial warming, it is plausible that warmer source waters fed tropical upwelling zones during the mPWP. Given early reconstructions of atmospheric CO_2_ concentration higher than the pre-industrial^3^,^4^,^49^, which have been supported by more recent studies^50^, the lack of tropical SST warming in areas outside the upwelling zones has proven puzzling. This was one reason enhanced meridional ocean heat transport was suggested on the basis of reconstructed mPWP SST gradients^42^. This provided a way to explain higher concentrations of atmospheric CO_2_ (compared with pre-industrial) while at the same time preserving tropical SST stability outside the upwelling zones.

Understanding the thermal stability of the western equatorial Pacific (WEP) warm pool has been a driver of Pliocene palaeoceanographic research in general for many years. While the focus of these studies has been on the early Pliocene rather than the mPWP, insights may be transferable from one to the other (Box 2).

Wara *et al*.^51^ developed Mg/Ca-based sea surface temperature (SST) records from calcareous tests of surface-dwelling planktonic foraminifers. These time series span the Pliocene to Recent at Ocean Drilling Program Sites 847 in the eastern equatorial Pacific (EEP) and 806 in the WEP (Fig. 2). The records suggest stable temperatures in the WEP and a small surface-temperature gradient across the equatorial Pacific, particularly in the early Pliocene, much similar to a modern El Niño event with warmer-than-average SST in the EEP. This led to the concept of a *Permanent El Niño-like* state during the Pliocene, with concomitant warming in the EEP, suggesting a weak zonal atmospheric circulation^51^.

One study (ref. 48) used quantitative faunal assemblage techniques to estimate SST and found no compelling evidence that the WEP was different from that of present day. Since faunal techniques (calibrated to present day) have a maximum limit of ∼30 °C, and 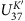 index becomes fully saturated at 28 °C, neither can document warming above modern warm pool conditions. Some late-Pliocene equatorial assemblages do show small non-analogue increases in thermophilic taxa that could be explained by brief periods with SST in excess of 30 °C (ref. 48).

O'Brien *et al*.^52^ and Zhang *et al*.^53^ addressed the issue of stability of the tropical warm pool during the *early* Pliocene by applying Mg/Ca, 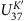 and TEX_86_ techniques. They found close agreement between 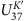 and TEX_86_ where they could both be applied, giving confidence to the application of TEX_86_ to the warm pool regions. The TEX_86_-based estimates of WEP SST were higher than previous studies based on Mg/Ca or faunal assemblages. They concluded that previous WEP Mg/Ca temperatures were underestimated because of changes in the Mg/Ca of Pliocene seawater (see next section for discussion of the Pliocene tropical Pacific and El Niño Southern Oscillation (ENSO)). It is important to note that WEP temperature reconstructions remain highly debated and no clear consensus has yet emerged within the palaeoceanographic community (see comments and counter comments on Zhang *et al*.^53^). This is perhaps unsurprising. Elevating atmospheric CO_2_ concentration to 400 p.p.m.v. (a 120-p.p.m. increase over pre-industrial) would create an additional radiative forcing capable of increasing tropical SSTs by 1–3 °C at most. Hence, the signal of change is small relative to the inherent uncertainties in SST reconstruction resulting in an unfavourable signal to noise ratio. Such scenarios of claim and counterclaim regarding tropical temperatures have been the subjects of significant discussion in other communities studying other time intervals. Overall, this highlights the need for further study, while at the same time anticipating divergent views, given the aforementioned signal to uncertainty ratio.

Besides documenting palaeoceanographic conditions during the mPWP, there have been many studies that focus on the terrestrial environment^10^,^16^,^19^. Work in the terrestrial realm is dependent on the heterogeneous distribution of localities where palaeoclimate signal carriers are preserved. Those outcrops and cores with suitable Pliocene chronology, or that contain continuous sequences, are few in comparison with the quantity of cores containing mPWP marine sediments retrieved by the International Ocean Discovery Program and its predecessors. Terrestrial proxies from near-shore marine cores allow for high-resolution continental–marine correlation of Pliocene palaeoclimate records (for example, refs 54, 55). Salzmann *et al*.^16^ summarized vegetation-based estimates of Pliocene (Piacenzian Stage) climate as being generally warmer and moister. Evergreen taiga, temperate forest and grasslands shifted northwards, which resulted in a reduction in tundra vegetation. Warm temperate forests spread in middle and Eastern Europe and tropical savannahs and woodland expanded in Africa and Australia, replacing deserts.

Arguably, the longest and most complete record of late-Pliocene high-latitude continental climate, including the mPWP, comes from Lake El'gygytgyn^56^, located in Northeast Arctic Russia in a basin formed ∼3.6 Ma by a meteorite impact. The many environmental proxies from the Lake El'gygytgyn core can be correlated to the LR04 stack and thus placed within the same chronostratigraphic framework as marine sequences (Fig. 2). The ability to correlate time series of terrestrial data with time series of marine data is rare in a Pliocene context because of the difficulties in assigning precise chronologies to most available terrestrial data. Lake El'gygytgyn records document polar amplification such as is seen in marine records^57^ and summer temperatures 8 °C warmer than present day, which persisted until ∼2.2 Ma. This supports other estimates of strong Arctic warming during parts of the late Pliocene, including the mPWP^58^.

Atmospheric concentration of CO_2_ during the Pliocene remains only partially constrained. A number of techniques exist to estimate CO_2_ (alkenones, B/Ca, *δ*^11^B, *δ*^13^C and leaf stomatal density), but the variability of estimates is high (Fig. 2). The majority of estimates indicate that CO_2_ concentration during the mPWP was higher than the pre-industrial; however, the increase above pre-industrial levels reconstructed from certain records is small and presents a challenge to attribute mPWP warmth to CO_2_ forcing alone^3^,^5^,^6^,^7^,^50^,^59^,^60^.

It is increasingly recognized that CO_2_ is just one agent of radiative forcing and that other greenhouse gases such as methane (CH_4_) are also important; however, these cannot be reconstructed at the current time for the Pliocene. Changes in continental features (including but not limited to orography and land cover) are also hypothesized to have increased long-term warmth. Furthermore, Unger and Yue^61^ have demonstrated the potential importance of atmospheric chemistry–climate feedbacks, as well as aerosols, in augmenting surface-temperature warming derived from a given increase in atmospheric CO_2_.

While palaeoenvironmental reconstructions have contributed greatly to understand the nature of the mPWP, the information available is insufficient to fully explain all aspects of the Earth's climate during this time.

## Palaeoclimate model simulations

Conceptual models of the Pliocene were first used to propose mechanisms and drivers of global and regional climate change demonstrated in proxy-based reconstructions^62^. For example, Crowley^63^ showed that the modest CO_2_ increase reconstructed for the mPWP was capable of producing a radiative forcing of ∼2 Wm^−2^, which may have been sufficient in explaining the warmth of the Pliocene globally, depending on the chosen Climate Sensitivity (the global mean temperature change in response to a given variation in atmospheric CO_2_ concentration). As discussed in the section above, such an approach could not explain the regional changes in reconstructed SST (based on planktonic foraminifera) that displayed little or no warming relative to present day in the tropics^18^,^20^. Therefore, an additional mechanism(s) working independently or combined with variations in CO_2_ concentrations in the atmosphere was required to drive and maintain the warmer conditions reconstructed for the mPWP^63^.

A likely additional driver of Pliocene warmth receiving attention in the early part of the 1990s, and still discussed today, is a change in ocean heat transport. This initially geologically driven hypothesis^42^ received further impetus from a conceptual modelling study published by Rind and Chandler^62^. Crowley^63^,^64^, based on a conceptual modelling framework, pointed out a paradox: a reduced latitudinal SST gradient implies potentially weaker atmospheric forcing of oceanic circulation, and hence weaker oceanic heat transport. Thus, the total benefit in terms of the ‘global net' heat transport from enhanced ocean heat transport was lower than would otherwise be expected^63^. This early conceptually based finding has recently been supported by the investigation of Pliocene ocean circulation and heat transport behaviour in climate models^65^.

**Figure 3:**
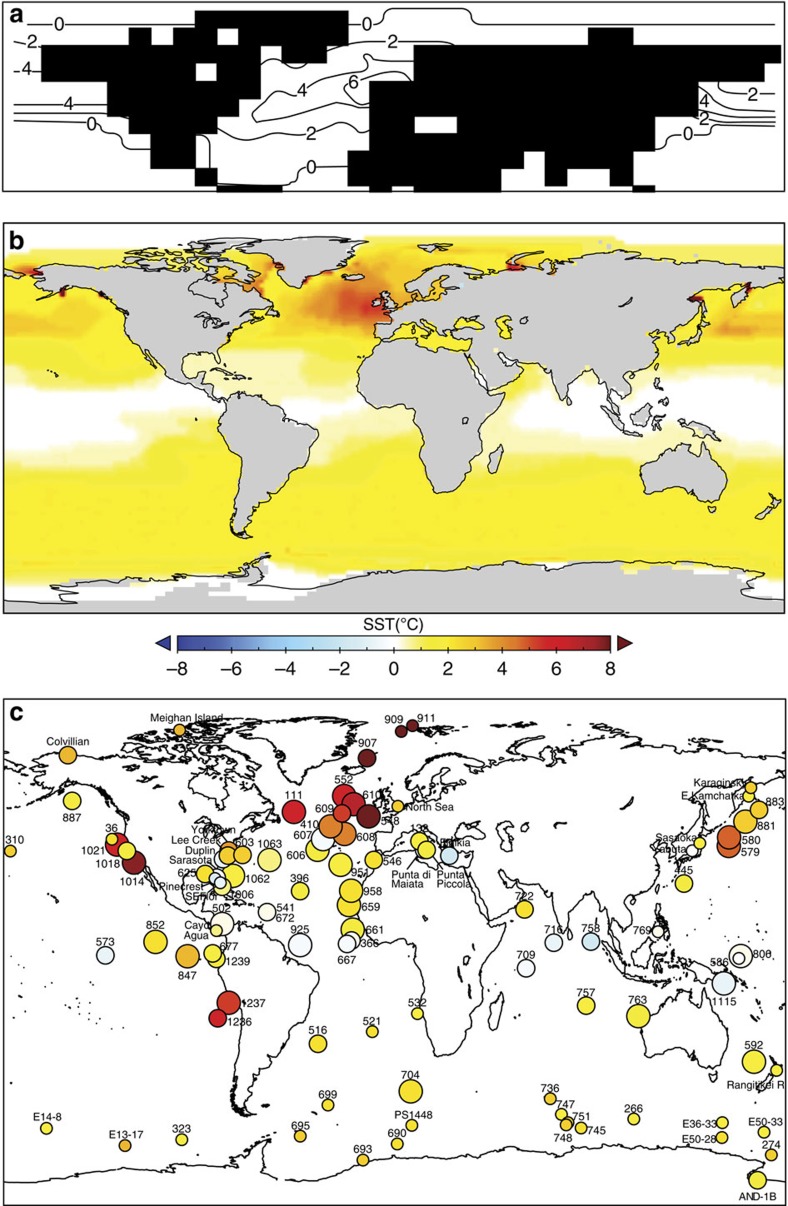
Evolution of the PRISM reconstruction. The PRISM mean annual SST anomalies. (**a**) PRISM0 Northern Hemisphere reconstruction^112^. (**b**) PRISM2 global reconstruction^20^,^21^. (**c**) PRISM3 confidence-assessed global reconstruction where larger diameter circles represent higher confidence levels; numbers designate sample localities^9^,^17^,^57^. SST anomaly colour scale the same for **b**,**c**.

**Figure 4:**
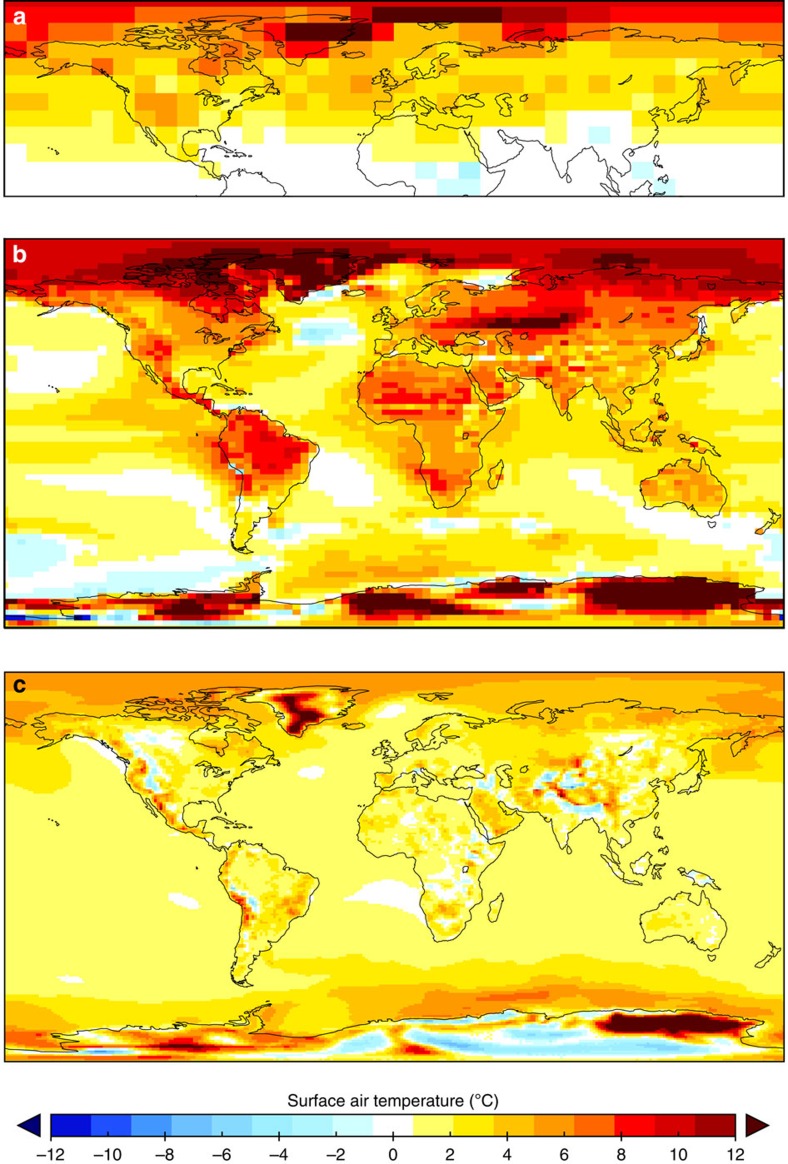
Evolution of climate simulations. Pliocene surface air temperature simulations. (**a**) Goddard Institute for Space Studies (GISS) Atmosphere-only climate model with PRISM prescribed boundary conditions^22^. (**b**) Hadley Coupled Climate Model Version 3 simulation initiated with PRISM2 boundary conditions^66^. (**c**) Community Climate Model Version 4 simulation initiated with PRISM3D boundary conditions^113^.

Arguably, the true benefits of the synergy between models and data were first realized in early fully numerical simulations of the mPWP climate using atmosphere-only General Circulation Models (AGCMs). Such models required monthly SSTs and sea ice, as well as vegetation cover, to be prescribed (boundary conditions). The PRISM palaeoenvironmental reconstruction provided the necessary data to underpin these boundary conditions that were held constant for the entire length of the model simulation. While this negated the possibility of examining atmosphere–ocean–vegetation feedbacks, it did provide a way to explore the response of the atmosphere, in isolation, to a given set of geological boundary conditions that at the time represented state-of-the-art knowledge about the mPWP. The first of these simulations used the PRISM0 reconstruction and focused on responses in the Northern Hemisphere^22^. Subsequent studies simulated the entire globe and used updated boundary conditions as more palaeoenvironmental information became available^23^,^24^. The current PRISM reconstruction (PRISM3D^17^) has been incorporated into a number of AGCMs as part of the Pliocene Model Intercomparison Project (PlioMIP; Box 3 and Figs 3 and 4).

With the evolution of the PRISM data sets and advances in modelling, AGCM simulations forced with PRISM boundary conditions enabled improvements in understanding the nature of the mPWP. AGCM simulations showed an increase in the global annual mean surface air temperature warming of 1.4–3.6 °C, relative to the pre-industrial^11^. Warming was greatest at high latitudes; consequently, the equator to pole temperature gradient decreased relative to modern. Surface air temperature increases were greatest in winter, as decreased snow and sea ice triggered a positive albedo temperature feedback. At low latitudes, temperatures were mostly unchanged, except for cooling over Northern Africa^22^,^23^,^24^. This model-predicted cooling was supported by palaeobotanical data and, in the simulations, was a response to a weakening and/or broadening of the Hadley Cell, which caused local subtropical clouds and evapotranspiration rates to increase. The hydrological cycle intensified, where regionally annual evaporation, rainfall and soil moisture all increased^22^,^23^,^24^.

Owing to the importance of potential ocean feedbacks on the climate that could not be simulated in AGCMs using prescribed SSTs (and the fact that prescribed SST experiments are often not in energy balance at the top of the atmosphere), modelling strategies quickly evolved to include a simplified slab ocean capable of predicting SST and sea-ice change. Atmosphere-slab ocean models were also employed to explore the sensitivity of Pliocene climate to atmospheric CO_2_ concentration (Chandler, unpublished data), as well as the sensitivity of climate predictions to orbital forcing^66^.

A major breakthrough was achieved through the use of fully coupled atmosphere–ocean General Circulation Models (AOGCMs), where the full three-dimensional response of the oceans was, for the first time, predicted. This milestone changed the relationship between models and marine data and moved the use of marine data away from providing model boundary conditions and towards providing verification data for model evaluation. For the first time, researchers explored the relative role of the atmosphere, oceans and cryosphere in contributing towards Pliocene warmth^67^. In contrast to AGCM experiments, and available geological archives, surface temperatures warmed across the globe, including in the tropics. Analysis of the predicted ocean circulation indicated reduced outflow of Antarctic bottom water, a shallower depth for North Atlantic deep-water formation and slightly weaker thermohaline circulation. Neither the oceans nor the atmosphere transported significantly more heat in the Pliocene scenario, and a significant driver of warmth was the reduced extent of high-latitude terrestrial ice sheets and sea-ice cover, resulting in a strong ice albedo temperature feedback^67^. This result countered the then-predominant view based on the geological record that there was an increase in ocean heat transport during the mPWP^42^.

Increasing utilization of AOGCMs unquestionably represented a paradigm shift in climate modelling for the mPWP and underpinned a host of further applications exploring, for example, the sensitivity of climate to greenhouse gas concentrations^68^,^69^,^70^,^71^, the importance of ocean gateways and ocean bathymetry^72^,^73^,^74^,^75^, Climate Sensitivity and longer-term sensitivity—Earth System Sensitivity^60^, the importance of uncertainties in model parameterization^76^,^77^, as well as detailed studies exploring the importance of individual boundary conditions in driving the warmth and polar amplification^78^. These individual studies enhanced our understanding of the mPWP. It is additionally important to understand the degree to which model predictions may differ. To this end, the PlioMIP was initiated in 2008 (Box 3). This provided the framework for multiple models to undertake an experiment with the same boundary conditions and has been a major driver in developing the relationship between data and models (see section on data/model comparison).

In addition, Earth System Models (models capable of including a variety of additional Earth system processes) are allowing entirely new components of the Pliocene climate system to be explored. They present their own unique challenges in terms of providing adequate geological constraints for the models. Earth System components have included interactive vegetation, allowing vegetation/climate feedbacks to be incorporated within simulations (for example, ref. 79).

Ice sheet models are now being used to better understand the validity of ice sheet reconstructions used in climate models^13^,^80^,^81^,^82^. Geological evidence suggests that the sea level could have been ∼20 m higher than present day during the mPWP^15^,^83^,^84^,^85^, which implies that there would likely have been a reduction in the size of the Greenland, West Antarctic and potentially the East Antarctic ice sheets. Proximal evidence for ice sheet size^86^,^87^ is too sparse around the ice sheet margins to enable detailed reconstructions of the extent of the major ice sheets. Therefore, our current understanding of plausible mPWP ice sheet extents is based on predictions using climate-forcing from a single climate model to force an offline ice sheet model (for example, refs 13, 88, 89, 90, 91, 92, 93, 94, 95). The multitude of reconstructions for mPWP ice sheets has highlighted the key areas of model disagreement. One example surrounding predictions of the East Antarctic ice sheet has sparked much debate, as some results indicate a modern grounding line^88^, while others imply more wide-scale retreat^13^,^96^. There is a general lack of evidence to provide constraints for mPWP ice sheet modelling, although there are interesting new data for the Pliocene, indicating a dynamic behaviour at the margins of East Antarctica^12^ and also thickening of the Antarctic interior^97^.

It is important to quantify the extent to which model predictions for ice sheets in the mPWP differ and as such the period has been used as a test bed for understanding the model dependency of ice sheet predictions during a warm interval in Earth History. Results from the Pliocene Ice Sheet Model Intercomparison Project (PLISMIP^98^) have shown that simulations using the same climate-forcing fields but different ice sheet models are relatively similar over Greenland^93^; however, the predicted details of ice configuration over West Antarctica and the subglacial basins of East Antarctica can differ^99^. *A priori* assumptions necessary to initiate a climate model (for example, ice configuration) and also the choice of model have the largest impact on the simulated Greenland ice sheet for the mPWP^81^,^93^. Important steps towards understanding model dependency have been made, and this has informed future iterations of palaeogeographic boundary conditions for PlioMIP Phase 2 (ref. 100). Using a compilation from the PLISMIP results and taking into account available geological constraints, the latest Pliocene palaeogeography contains ice sheet configurations where there is highest confidence in the possibility of ice presence during the warmest parts of the Pliocene. The PRISM4 palaeogeography is more internally consistent than the previous PRISM3 topographic reconstruction^101^ as it also takes into account the glacial isostatic response of loading specific Pliocene ice sheets (for example, ref. 102) and includes components such as the contribution of dynamic topography caused by changes in the mantle flow (for example, ref. 103).

Earth System Models incorporating representations of atmospheric chemistry are being used to simulate the effects of altered vegetation patterns on dust and aerosol emissions and the radiative forcing of such agents that have little or no expression within geological archives. For example, Unger and Yue^101^ calculated terrestrial ecosystem emissions and atmospheric chemical composition for the mPWP and the pre-industrial era. Tropospheric ozone and aerosol precursors from vegetation and wildfire were ∼50% and ∼100% higher in the mPWP because of the spread of the tropical savanna and deciduous biomes, respectively. The chemistry–climate feedbacks contributed to a net global warming that is +30–250% of the carbon dioxide effect, and a net aerosol global cooling masked 15–100% of the carbon dioxide effect. Although an exciting development, this realization presents a huge challenge to the palaeoclimate data community to develop proxies for atmospheric chemistry and aerosols. Such studies highlight the emergence of new unknowns in terms of atmospheric forcing in the Pliocene that are potentially more important than the currently known uncertainty in CO_2_ forcing, which from a modelling point of view can be investigated through CO_2_ sensitivity experiments.

Rapid progress has been made since the emergence of palaeoclimate model simulations for the mPWP. Models have incorporated geological data in the form of boundary conditions, but have also been used in isolation to understand particular climate mechanisms. We suggest that modelling results are most powerful when they are constrained by geological data.

## Data/model comparison

**Figure 5:**
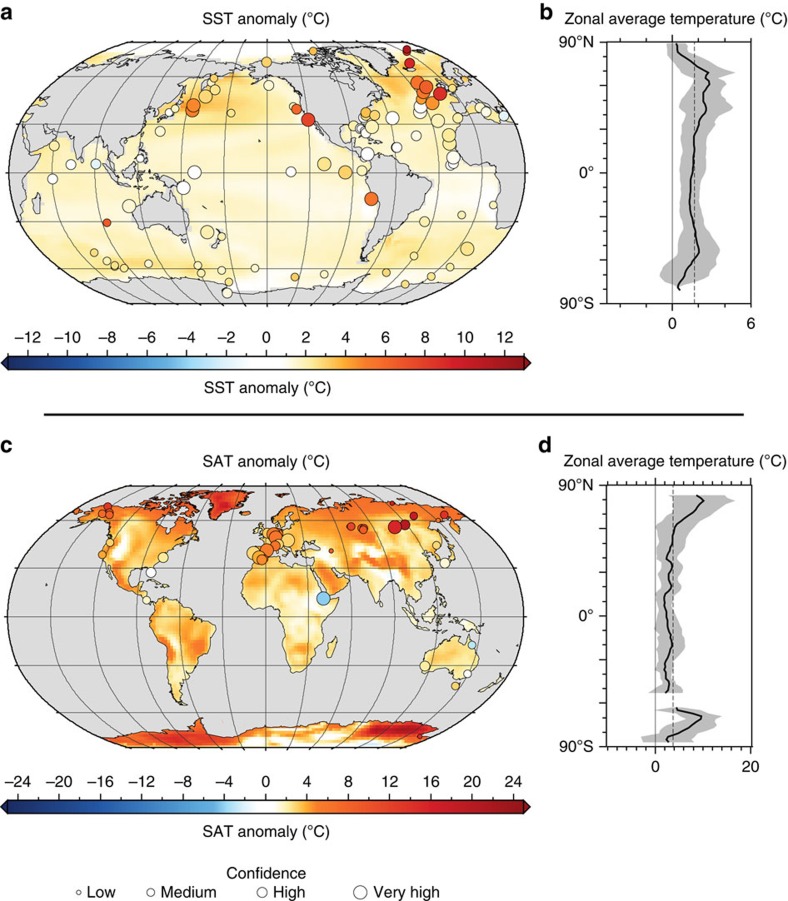
Comparison of Data and Models. International Panel on Climate Change (IPCC) data model comparison. Comparison of PRISM proxy data and the PlioMIP multimodel mean (MMM) simulation, (**a**) circles are PRISM SST anomalies, (**b**) zonally averaged PlioMIP MMM SST anomalies, (**c**) circles are PRISM land surface air temperature (SAT) anomalies, (**d**) zonally averaged MMM SAT anomalies. Zonal MMM gradients (**b**,**d**) are plotted with a shaded band indicating 2*σ*. Site-specific temperature anomalies estimated from PRISM proxy data are calculated relative to present site temperatures and are plotted (**a**,**b**) using the same colour scale as the model data, and a circle-size scaled to estimates of data confidence^9^,^10^,^11^. Modified from Box 5.1, Fig. 1,^83^.

When performing any kind of climate model simulation, it is important that the model be compared and evaluated against available data. Where data and models agree on the degree of temperature change (for example), there can be more confidence in the performance of that model. The utilization of AOGCMs in the context of the mPWP rapidly transformed the role of geological archives of surface temperature from providing model boundary conditions to providing the means to independently evaluate model performance. This was an important step as it facilitated a new way to use the Pliocene to evaluate the same models that are used to predict future climate change (Fig. 5).

To date, all mPWP data/model comparisons have used surface-temperature data from the PRISM palaeoenvironmental reconstructions. These data, representative of a time slab, have been compared with equilibrium (snapshot style) climate model simulations (for example, refs 9, 10, 11, 41, 57, 67). Published data/model comparisons for the mPWP have identified many regions of agreement between data and model predictions, such as in the Southern Ocean (for example, ref. 11; Fig. 5). They have highlighted potential inconsistencies between data and models in the tropics, where models may predict surface temperatures that are too warm compared with data^11^,^16^,^50^,^104^,^105^ (Fig. 5). Comparison with SSTs has suggested that the magnitude of warming predicted by models in the North Atlantic may be insufficient^10^,^57^, and that polar amplification may be too weak^10^,^57^,^58^ (Fig. 5). As discussed in Box 1, the averaging of geological ‘interglacial' climate signals across the mPWP makes these initial observations regarding model performance unclear.

At present, an assessment of suggested model shortcomings against uncertainties in the interpretation of surface-temperature records from geological data can only conclude that the implications of these patterns of data/model discord remain uncertain, and there are several reasons to support this view.

PlioMIP has shown a substantial spread in model predictions (Box 3). Therefore, previous studies that have analysed the performance of a single model and have suggested that data/model comparison is able to demonstrate a structural weakness in climate models may be too simplistic^11^. Forcings and model experimental design are at best incomplete, or at worst incorrect. This covers an uncertainty in the agents of radiative forcing (including atmospheric greenhouse gas composition, chemistry/climate feedbacks)^61^, ice sheet configuration^81^ and the likelihood that similarities drawn between modern and mPWP topography, drainage and bathymetry are overstated. Finally, the interpretation of surface-temperature patterns derived from geological archives are evolving (for example, refs 52, 53; Box 1).

The current debate regarding mPWP (and Pliocene in general) stability of tropical SSTs is a key illustration of this point. Recent re-evaluations of the Mg/Ca data incorporate the effects of changing concentrations of Mg/Ca in the water column through time. This, as well as the application of a newer proxy (TEX_86_), has indicated that surface ocean temperatures in the WEP warm pool may have been 1–2 °C higher than modern^52^. If true, this would effectively resolve the mismatch between tropical Pacific Ocean temperature data and predictions of AOGCMs for the mPWP^52^ (Fig. 5).

We have learned much about the environment of the mPWP and our ability to reproduce it with climate models. Data/model comparison is complex and requires a full and open discussion of (a) the inherent strengths and weaknesses of climate models, (b) uncertainties in geological boundary conditions used within models to facilitate simulations of the mPWP and (c) uncertainties in the geological archives and their interpretation. A key challenge for the future is to quantify and reduce uncertainty in both palaeoenvironmental reconstruction and climate modelling. In this way, the mPWP can achieve its potential in terms of informing us about processes and the key mechanisms associated with global warming, and will be better placed to inform us about the likely long-term environmental and climate effects of higher than pre-industrial concentrations of atmospheric CO_2_ (through more accurate assessments of Climate and Earth System Sensitivity).

## Outlook

We have provided evidence that synthesis of observations and models has enhanced understanding of the mPWP. We also maintain that one of the fundamental lessons from previous data/model comparisons (see Box 1) is the need to make palaeoenvironmental reconstructions and climate model simulations more consistent in time.

Our ability to correlate and date multiple marine records using the LR04 timescale has limits. There is uncertainty in correlation of particular events (for example, Marine Isotope Stages) primarily because of the character of the local isotopic record and the assumption of linear sedimentation rates. In addition, ice sheet response times, tuning errors and uncertainty in the Laskar^106^ orbital solution are all potential sources of error. Thus, near 3.0 Ma temporal uncertainty could be as much as 6–15 ky (ref. 29). Global average sediment accumulation rates vary, on average, between 3 and 5 cm ky^−1^. Bioturbation effect (mixing of sediment due to biologic activity) generally ranges between 4 and 8 cm depth from the sediment–water interface. Consequently, the benthic *δ*^18^O signal (and any other palaeoclimate signal recorded in deep-ocean sediments) is time-averaged and temporal homogenization could range from 0.8 to 2.5 ky.

Terrestrial sequences are generally more difficult to date. Age determination relies on the presence of materials compatible with isotopic dating systems, magnetic reversals and/or faunal and floral zonations calibrated to age. Lake settings sometimes show near continuous sedimentation, and geophysical logs (for example, magnetic susceptibility) can often be tuned to orbital forcing, providing an age model as accurate as the marine records, allowing direct marine–terrestrial correlation. These records are generally scarce, and the terrestrial realm is in all but a few unique sedimentary settings not going to be as completely reconstructed as the marine palaeoenvironment. Thus, terrestrial reconstructions are going to carry a greater degree of diachrony than marine reconstructions.

**Figure 6:**
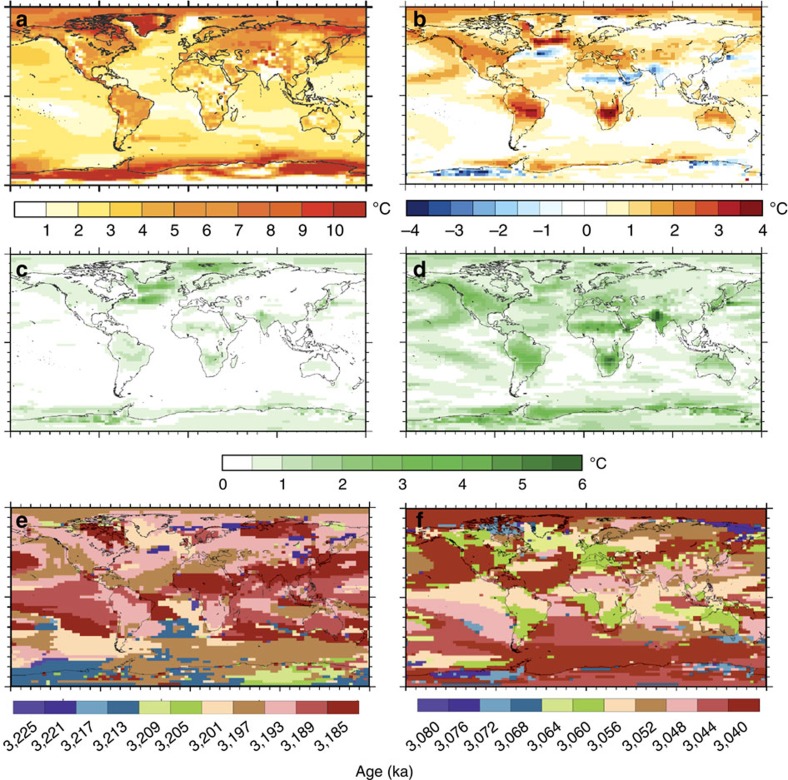
Simulating Pliocene climate variability. Hadley Coupled Climate Model Version 3 (HadCM3) simulations of surface air temperature (SAT °C) variability during the Pliocene redrawn and modified from ref. 107. (**a**) Annual mean Pliocene SAT (°C) prediction for interglacial Marine Isotope Stage (MIS) KM5c minus a pre-industrial experiment. (**b**) Annual mean SAT for Interglacial MIS K1 minus KM5c. Note that MIS K1 shows warmer SATs in most regions of the world compared to KM5c. (**c**) Variability in SATs 20 Kyrs ± of the benthic oxygen isotope peak of the KM5c interglacial. (**d**) Same as **c** but showing SAT variability ±20 Kyrs around the benthic oxygen isotope peak of the K1 interglacial. Note the larger degree of SAT variability in **d** compared to **c**. (**e**) Timing of the maximum SAT warming relative to the pre-industrial in each model grid box for KM5c ± 20 Kyrs. (**f**) Same as **e** but for K1 ±20 Kyrs. **e** and **f** Maximum SAT warming relative to the pre-industrial is not globally synchronous and varies in nature between MIS KM5c and K1. Modified from ref. 107.

A recent study^107^ demonstrated that orbitally forced changes in surface air temperature during interglacial events within the mPWP can be substantial and differ from one interglacial to another. Thus, uncertainties in correlation could easily contribute to patterns of apparent data/model disagreement. This is especially likely if geological archives (for example, the higher latitudes) preserve a growing season signal rather than the mean annual temperature (discussed in palaeoenvironmental reconstruction section). Climate model results indicate that peak surface temperatures associated with MIS KM5c and K1 interglacials were not globally synchronous, highlighting leads and lags in temperature in different regions. Furthermore, variability of surface temperature ±20 kyr surrounding MIS KM5c was 2–3 °C, but for K1 locally exceeded 5 °C. This indicates that the choice of the time interval is critical to the potential success of data/model comparison (Fig. 6).

The development of high-resolution proxy data time series within the mPWP (see Box 1) is an important step in characterizing climate variability at a local level over orbital timescales. Recognition and careful evaluation of stratigraphic factors described above will be necessary to objectively choose those marine sequences that can be confidently correlated to events such as KM5c. Performing sensitivity experiments with climate models, in which characteristics of orbital forcing are changed appropriately to any given time point, will be important in examining patterns of climate change recorded in geological time series. With complex and more computationally demanding climate models, this is likely to take the form of a series of snapshot-style experiments where the orbital forcing is varied appropriately to the time point in question and run to equilibrium. Another approach towards characterizing mPWP climate variability is to use computationally efficient, although lower (spatial) resolution-transient simulations. Experiments with time-dependent orbital forcing as well as dynamic ice sheets and vegetation^108^ provide estimates of the mean annual global warming and insight into feedbacks.

Not every warm peak within the mPWP has equal relevance in understanding future climate change, since specific warm intervals ‘interglacials' occurred under conditions of orbital forcing, unlike today. Therefore, it is important to either separate out the effect of orbital forcing on Climate Sensitivity or target a time period in which the orbital configuration is similar to modern within the mPWP. In the context of longer-term Climate Sensitivity (Earth System Sensitivity^11^,^108^,^109^), we must acknowledge that the effect of prior orbital forcing would continue to be present in parts of the Earth system, such as the ice sheets and deep-ocean temperatures because of their response times and memory effects.

Climate modelling is enabling the mPWP community to consider, in a more robust way, new intervals for data acquisition and reconstruction, at the same time narrowing the relevant time window. In the near future, models and data will be more ‘time consistent', meaning that models can be given more appropriate forcings to better represent the narrower time interval being reconstructed by geologists^110^. This means that orbitally induced surface-temperature variability will have a reduced potential to bias data/model comparisons. Even so, limitations in correlation will continue and climate variability at suborbital timescales will remain an important factor.

In the future, the integration of mPWP data and climate model simulations will be enhanced by (1) establishing a more precise chronology of the proxy data, (2) creating holistic regional and locality-based palaeoenvironmental reconstructions, which can be compared directly to simulated environments produced through proxy-modelling components (for example, isotope-enabled AOGCMs, predictive plankton and vegetation models and so on) of Earth System models, (3) providing climate models with boundary conditions that are more consistent with the intervals of time studied for environmental reconstruction (including more confident greenhouse gas-forcing calculations) and (4) producing climate ensembles and transient climate simulations that more adequately capture orbital and environmental variability around any studied interval of the mPWP.

## Additional information

**How to cite this article:** Haywood, A. M. *et al*. Integrating geological archives and climate models for the mid-Pliocene warm period. *Nat. Commun.* 7:10646 doi: 10.1038/ncomms10646 (2016).

## Figures and Tables

**Figure 1:**
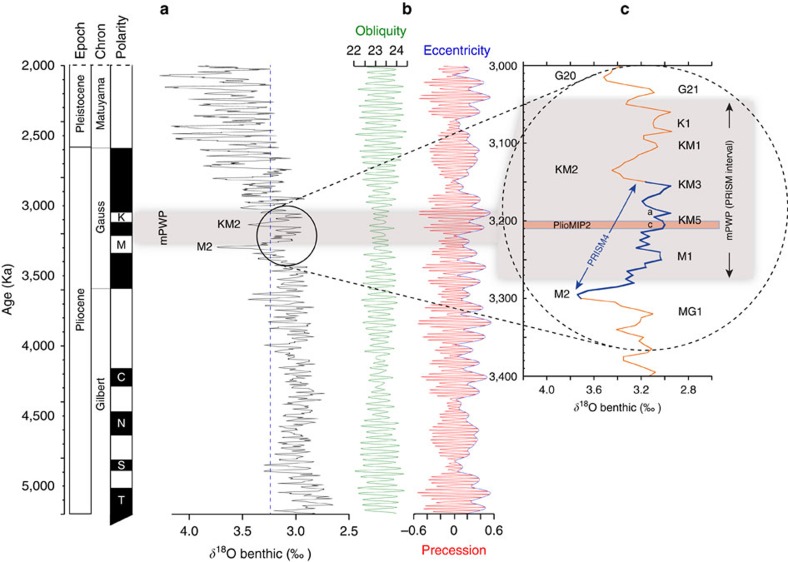
The Pliocene Research Interpretation and Synoptic Mapping Project Interval. The PRISM interval in relation to the long-term climate evolution of the late Pliocene. (**a**) LR04 benthic oxygen isotope stack and timescale of Lisiecki and Raymo^29^. Vertical dashed line shows present day *δ*^18^O value. The mPWP or PRISM3 warm interval (3.264–3.025 Ma) is shown by the horizontal shaded grey bar. (**b**) Laskar *et al*.^106^ values for obliquity (°), eccentricity and precession. (**c**) Details of LR04 timescale for the mPWP and position of PRISM4 and PlioMIP2 focus. Positions of Marine Isotope Stages MG1, M2, M1, KM5, KM3, KM2, KM1, K1, G21and G20 are shown.

## Pliocene Research Interpretation and Synoptic Mapping

The initial PRISM reconstruction was confined to the North Atlantic region. Palaeotemperature estimates were based on quantitative analysis of foraminifer and ostracode faunas from deep-sea cores and continental sections. Even this early and relatively primitive analysis documented a level of polar amplification of surface temperatures unseen today^42^. With the addition of marine data from the North Pacific and for the first time vegetation or land surface cover, sea level and ice volume/distribution estimates, PRISM was extended to a Northern Hemisphere Reconstruction^112^. Since that time each successive phase of the project (PRISM1 through 3) has added a new aspect of the palaeoenvironment or refined existing data sets^16^,^17^,^18^,^19^,^20^,^21^.

As the magnitude of the Pliocene temperature anomaly became better defined, new methods of standardization (for example, calibration of various palaeothermometres to the same modern temperature data set) and refinement of the chronologic limits of the PRISM time slab using newly developed oxygen isotope stratigraphy^29^, PRISM consistently improved the overall confidence of the reconstruction^20^,^21^.

The PRISM3D global reconstruction consists of six discrete data sets representing different aspects of the Pliocene world: sea surface temperatures, deep-ocean temperatures, land vegetation, ice distribution, topography and sea level^17^. It differs from its predecessors in that it includes a deep-ocean temperature reconstruction^114^, integrated geochemical sea surface temperature proxies to supplement faunal and floral-based estimates and uses numerical models for the first time to augment palaeobotanical data in the creation of a biome-based land cover scheme^16^.

The chronostratigraphic framework within which a palaeoenvironmental reconstruction is developed is fundamental to its uncertainty. When the PRISM project commenced, confident correlation of sequences from one ocean basin to another was limited by the resolution of the palaeomagnetic timescale and temporally calibrated fossil evolutionary first and last appearance events. This limitation and initial observations from the North Atlantic reconstruction defined a 300,000-year time slab, the PRISM interval, currently known as the mPWP, within which long-distance correlations were possible, and the warm phases of climate could be averaged. This, however, created a situation where non-coeval conditions were smoothed and compared, and a super-equilibrium climate was created that may never have existed. This has significant implications for the validity of data/model comparisons^110^,^115^ (see Data Model Comparison section for discussion on the importance of climate variability during the mPWP).

The PRISM4 reconstruction has new topography and ocean bathymetry, ice sheets, sea level, soils and lake data sets. SST and BIOME land cover remain the same as in PRISM3. The PRISM4 reconstruction represents the most complete and internally consistent global-scale conceptual model of the Pliocene. New high-resolution time series are being developed with millennial-scale resolution to provide information on regional variability. These time series use multiple factors (for example, T, S, productivity, precipitation, diversity, nutrients and so on), allowing for a more holistic and nuanced description of the palaeoenvironmental setting and, where possible, will have a strong marine–terrestrial correlation. These elements of the PRISM4 reconstruction will be best suited for evaluation of new climate model simulations that will be carried out as part of the second phase of PlioMIP (PlioMIP2). PRISM4 data can be found here http://geology.er.usgs.gov/egpsc/prism/index.html.

## Character and dynamics of Pliocene climate

Since the first assessments of Pliocene environments, the key characteristics of the spatial patterns of climate change have emerged that provide important insights into the dynamics of warm-climate states during the Pliocene. The first key characteristic to emerge was the meridional profile of surface-temperature change^18^,^35^,^42^. Both marine and terrestrial proxy reconstructions clearly illustrated a progressive enhancement of the warming signal towards the high latitudes, resulting in a reduced equator to pole temperature gradient^16^,^19^,^35^. Besides being a primary feature of the mPWP, this character has also been demonstrated within the early Pliocene^116^. This is important for a number of reasons. First, it points to a clear polar amplification of the warming signal and underlines the significance of ice albedo and potentially cloud albedo feedbacks at the higher latitudes^67^,^117^. In addition, the equator to pole temperature gradient is a first-order control on the position and intensity of atmospheric pressure systems, modes of climate variability such as the North Atlantic/Arctic Oscillation, the position and intensity of westerly wind belts and Hadley circulation. Changes in the Hadley circulation driven by reductions in the meridional temperature gradient have been suggested as a primary driver in changes to the character of precipitation in the tropics (for example, ref. 22). Climate models have demonstrated that the ascending branch of the Hadley cell expands polewards in the Pliocene, implying a weakening of the meridional circulation in response to the warmer SST in the higher latitudes^22^,^23^,^24^. In North Africa, pollen-based reconstructions of vegetation point to a reduction in arid deserts and an extension of Savannah-type environments^16^,^19^. This has been linked to alterations in Hadley circulation that led to regional increases in total precipitation, thereby promoting surface-temperature cooling through enhanced evaporation and latent heat flux^22^,^23^,^24^.

A reduced zonal temperature gradient in the tropics is a second key characteristic to emerge. While this has been demonstrated for the mPWP, it is most clearly expressed in the early Pliocene. Time series analyses of SST trends identified a substantially reduced zonal temperature gradient in the tropical Pacific, with relative temperature stability in the western Pacific (to within 1 or 2 °C of modern SSTs), and a clear pattern of warming in the eastern Pacific^50^,^51^,^116^. This zonal temperature pattern has similarities to the pattern associated with El Niño events, yet it apparently persisted for millions of years, leading to the description of this pattern of zonal temperatures as a permanent El Niño-like (or El Padre) state^51^. The zonal temperature gradient in the tropics is a key constraint on the strength of the Walker circulation (weaker in the Pliocene) where parcels of air follow a closed circulation in the zonal and vertical directions^51^,^52^,^116^. This circulation is driven by differences in heat distribution between ocean and land and interacts with the Hadley Circulation^51^. The reduced zonal temperature gradient in the tropics may have important implications for the behaviour of the El Niño Southern Oscillation (a primary mode of climate variability in the tropics and extra tropics), with climate models suggesting that El Niño events during the Pliocene may have been more persistent, frequent or stronger^70^. Indeed, recent work has focussed on the development of a unified theory to account for the emergence of reduced zonal temperature gradients as well as meridional temperature gradients in the Pliocene, that may be relevant for understanding climate states beyond the Pliocene^116^.

## The Pliocene Model Intercomparison Project

To understand uncertainty in model predictions and how this affects patterns of data/model agreement and disagreement, multiple AGCM and AOGCMs have been applied to the mPWP in a fully coordinated way through the PlioMIP. This, as much as the first use of AOGCMs, has served to reshape our thinking and understanding of Pliocene climate, and has rapidly driven forward the frontier of Pliocene climate modelling. For the first time, PlioMIP facilitated an exploration of robust model predictions^11^ (predictions that are reproduced by a number of models rather than a product of a single model).

PlioMIP utilized the PRISM3D palaeoenvironmental reconstruction to provide a consistent set of boundary conditions to the climate models and most importantly within a common experimental framework^100^. PlioMIP provided new methodological rigour towards the understanding of large-scale features of the Pliocene climate and climate sensitivity^11^, patterns of ocean circulation and monsoon behaviour^65^. Results from PlioMIP identified consistency in surface-temperature change across models in the tropics, but showed a lack of consistency identified in model responses at high latitudes. In contrast, model predictions were shown to be inconsistent in terms of total precipitation rate in the tropics^11^. Model-predicted global annual mean surface temperatures increased by 1.84–3.6 °C and show a greater range when using AOGCMs than using AGCMs^12^. There was no clear signal in model predictions to support enhanced Atlantic Meridional Overturning Circulation and Ocean Heat Transport to the high latitudes^65^. Analysis demonstrated that clear sky albedo and greenhouse gas emissivity dominate polar amplification of surface-temperature warming during the Pliocene^117^. This demonstrated the importance of specified ice sheet and high-latitude vegetation boundary conditions and simulated sea ice and snow albedo feedbacks^117^ The simulated weakened mPWP East Asian winter winds in north monsoon China and intensified East Asian summer winds in monsoon China agreed well with geological reconstructions^54^.

For the first time, PlioMIP systematically assessed the ability of models to reproduce patterns of regional temperature change recorded by geological archives. Data were used to identify potential weaknesses in model predictions^9^,^10^,^11^, but also highlighted potential deficiencies in interpretation of geological data and inconsistencies in the methodology for data/model comparison (discussed further in Outlook section and Box 1). While current limitations in age control and correlation make interpreting data/model discrepancies challenging^9^,^10^,^107^,^110^, comparison with both sea-surface and surface-air temperature proxies indicates that climate models potentially underestimate the magnitude of polar amplification^9^,^10^,^57^.

The second phase of PlioMIP (PlioMIP2) has been launched using state-of-the-art PRISM4 geological boundary conditions to constrain new climate model simulations^100^. In PlioMIP2, a suite of experiments will be performed to address our understanding of the warm Pliocene as well as its utility in the context of future climate change. The temporal focus for PlioMIP2 has been matched to the data collection strategy adopted by PRISM4 to further enhance the connection between models and data and to improve the validity of future data/model comparisons. PlioMIP2 information can be found here: http://geology.er.usgs.gov/egpsc/prism/7_pliomip2.html.
